# Histologic analysis and lipid profiling reveal reproductive age-associated changes in peri-ovarian adipose tissue

**DOI:** 10.1186/s12958-019-0487-6

**Published:** 2019-06-12

**Authors:** Shweta S. Dipali, Christina R. Ferreira, Luhan T. Zhou, Michele T. Pritchard, Francesca E. Duncan

**Affiliations:** 1 Department of Obstetrics and Gynecology, Feinberg School of Medicine, Northwestern University, 303 E. Superior Street, Lurie 7-117, Chicago, IL 60611 USA; 2 Center for Analytical Instrumentation Development, Department of Chemistry, Purdue University, West Lafayette, IN USA; 3 Bindley Bioscience Center, Purdue University, West Lafayette, IN USA; 4 Department of Pharmacology, Toxicology & Therapeutics, University of Kansas Medical Center, Kansas City, KS USA

**Keywords:** Adipose, Peri-gonadal, Triacylglycerol, Ovary, Aging

## Abstract

**Background:**

Reproductive aging is a robust phenotype that occurs in all females and is characterized by a significant reduction in gamete quantity and quality, which can have negative consequences on both endocrine function and fertility. Age-associated differences in the oocyte, follicle, and ovary have been well-documented, but how the broader environment changes with age is less well understood. Fat is one of the largest organs in the body, and peri-gonadal adipose tissue surrounds the rodent ovary and comprises a local ovarian environment. The goal of this study was to characterize how peri-ovarian adipose tissue changes with advanced reproductive age.

**Methods:**

We isolated peri-gonadal adipose tissue from two cohorts of CB6F1 mice: reproductively young (6–12 weeks) and reproductively old (14–17 months). A comparative histological analysis was performed to evaluate adipocyte architecture. We then extracted lipids from the tissue and performed multiple reaction monitoring (MRM)-profiling, a mass spectrometry-based method of metabolite profiling, to compare the lipid profiles of peri-gonadal adipose tissue in these age cohorts.

**Results:**

We found that advanced reproductive age was associated with adipocyte hypertrophy and a corresponding decrease in the number of adipocytes per area. Of the 10 lipid classes examined, triacylglycerols (TAGs) had significantly different profiles between young and old cohorts, despite quantitative analysis revealing a decrease in the total amount of TAGs per weight of peri-gonadal adipose tissue with age.

**Conclusions:**

These findings pinpoint age-associated physiological changes in peri-gonadal adipose tissue with respect to adipocyte morphology and lipid profiles and lay the foundation for future studies to examine how these alterations may influence both adipocyte and ovarian function.

**Electronic supplementary material:**

The online version of this article (10.1186/s12958-019-0487-6) contains supplementary material, which is available to authorized users.

## Background

Aging is characterized by progressive physiological deterioration at both the cellular and tissue levels. This deterioration causes impaired function and is one of the primary risk factors for many human diseases [1]. The female reproductive system is unique in that it is the first organ system in the body to age. Female reproductive aging is marked by decreased egg quality and quantity that results in decreased fertility and a loss of endocrine function [2]. Women of advanced reproductive age have a higher risk of miscarriage and/or of having offspring with genetic defects, which is a growing problem as more women worldwide are delaying child-bearing [3, 4].

The age-associated decline in gamete quality is multi-factorial and has been attributed to changes at the level of the gamete, the follicle, and the immediate stromal environment. Reproductive aging in the oocyte is correlated with alterations in chromosomes, mitochondria, nucleoli, ribosomes, microtubules, spindle poles, and transzonal projections [5–11]. The oocyte does not develop in isolation but is instead highly dependent on surrounding granulosa cells within the intact ovarian follicle. Follicles isolated from reproductively old mice exhibit altered gene expression patterns and hormone profiles and contain oocytes with reduced meiotic competence, including a higher incidence of spindle defects, relative to reproductively young controls [6, 12]. Moreover, the growth of ovarian follicles is dependent on the stromal microenvironment which includes a highly structured extracellular matrix (ECM), fibroblasts, smooth muscle cells, endothelial cells, theca-interstitial cells, and immune cells. This ovarian microenvironment changes with advanced reproductive age and is associated with increased fibrosis, the presence of a unique population of multinucleated macrophage giant cells, and elevated expression of inflammatory cytokines [13]. How the physiology of the environment immediately surrounding the ovary changes with age has not been carefully examined.

In rodents, the ovary is surrounded by visceral fat pads in the peri-gonadal region [14, 15]. This periovarian fat depot does not exist in humans but is most highly analogous in function to the visceral omental fat [14]. Adipose tissue plays an important role in energy storage, temperature regulation, and mechanical protection, in addition to having several immune and endocrine functions [14]. The majority of cells in adipose tissue are adipocytes, which store energy as fat within lipid droplets, but fat tissue also contains pre-adipocyte progenitor cells, fibroblasts, endothelial cells, pericytes, and immune cells [16]. Aging is associated with a shift of adipose tissue from the periphery to a more central deposition, especially in the viscera, which contributes to increased metabolic disease in adults [17–19]. With age, fat tissue metabolism defects occur ranging from preadipocyte dysfunction to the inability of fat cells to properly store or release energy or respond to excess lipotoxic fatty acids [20]. Furthermore, aging is also associated with cellular senescence and chronic, low-grade inflammation in adipose tissue with increased local and circulating proinflammatory cytokines [20, 21].

The goal of our study was to examine whether changes in periovarian adipose tissue architecture and lipid profiles are detectable at a developmental window known to be associated with established reproductive aging phenotypes, including decreased ovarian reserve, increased egg aneuploidy, and increased ovarian stromal fibrosis [6, 8, 13, 22–25]. We expect that age-associated changes in adipose tissue may impact ovarian function given the established connections between lipids, the ovary, and the oocyte. For example, the composition and metabolism of lipids within ovarian follicles, both in follicular fluid and as lipid droplets within the oocytes, impacts the developmental competence of the oocyte [26, 27]. Moreover, the lipid profile in human follicular fluid changes with age [28]. Altered lipid profiles within the ovary are also characteristic of disease states. Ovaries from rabbits with hypothyroidism have increased total cholesterol and glycogen content, but reduced triacylglycerol content in comparison to control rabbits [29]. Moreover, hypothyroidism reduces the size of follicles in the rabbit ovary, in addition to inducing hypertrophy of adipocytes and infiltration of macrophages into periovarian adipose tissue, together suggesting interplay between the adipose tissue and the ovary [30].

Through a comparative histological analysis of peri-gonadal adipose tissue of reproductively young and old mice, we found that advanced reproductive age was associated with adipocyte hypertrophy and a corresponding decrease in the number of adipocytes per area. We then used multiple reaction monitoring (MRM)-profiling, a mass spectrometry based method of metabolite profiling that allows accelerated discovery of a large number of discriminating molecular features, to compare the lipid profiles of peri-gonadal adipose tissue from reproductively young and old mice [31, 32]. Of the 10 lipid classes examined, triacylglycerols (TAGs) had significantly different profiles between young and old cohorts. Interestingly, however, quantitative analysis revealed that there was a decrease in the total amount of TAGs per weight of peri-gonadal adipose tissue with age. Overall, this study pinpoints key physiological differences that occur with age in peri-gonadal adipose tissue and sets the stage for future studies to explore the consequences of such changes on reproductive aging, especially given the close proximity of the adipose tissue to the ovary and oocytes.

## Methods

### Animals and peri-gonadal adipose tissue collection

Reproductively young CB6F1 mice (6–12 weeks old) were obtained from Envigo (Indianapolis, IN) and reproductively old mice (14–17 months old) were obtained from the National Institute on Aging Aged Rodent Colony (National Institutes of Health, Bethesda, MD). Based on a linear extrapolation of age, the 14–17 month old cohort corresponds to women in their late thirties to early forties. Studies in several strains across multiple laboratories have demonstrated that female mice at this age exhibit reproductive aging phenotypes [6, 8, 10, 13, 22–25, 33]. Studies with CB6F1 mice within the same age cohorts demonstrate a significant age-associated decrease in the number of primordial follicles or ovarian reserve as well as an increase in egg aneuploidy [6, 8]. All mice were housed in a controlled barrier facility at Northwestern University’s Center for Comparative Medicine on the Chicago Campus under constant temperature, humidity, and light (14 h light/10 h dark). The mice in the different age cohorts were fed specific chows in their respective source facilities, but upon arrival to Northwestern University, water and Teklad Global irradiated 2016 chow containing minimal phytoestrogens and no soybean or alfalfa meal (Envigo, Madison, WI) were provided ad libitum (Additional file 1: Table S1). A summary of the fat content and fatty acid composition of the chows is listed in Additional file 1: Table S1. All animal experiments described here were approved by the Institutional Animal Care and Use Committee (Northwestern University) and were performed in accordance with National Institutes of Health Guidelines.

**Fig. 1:**
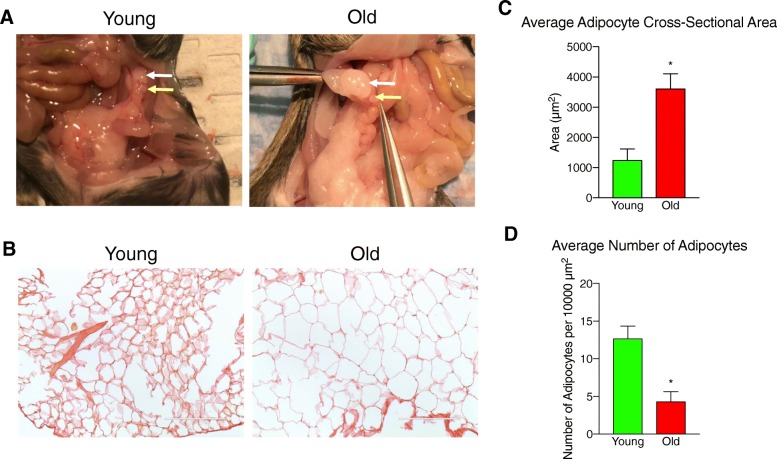
Adipocyte size in peri-gonadal adipose tissue increases with advanced reproductive age. **a** Representative anatomical images showing the ovary (yellow arrow) and the surrounding peri-gonadal adipose tissue (white arrow) in reproductively young and old mice. **b** Representative images of histological sections of peri-gonadal adipose tissue from reproductively young and old mice stained with PSR. The scale bars = 200 μm. **c** The average cross-sectional adipocyte area (μm2) in peri-gonadal adipose tissue from reproductively young (green) and old (red) mice. b The average number of adipocytes per 10,000 μm2 in peri-gonadal adipose tissue from reproductively young (green) and old (red) mice. Error bars represent standard error between samples. *P value ≤0.05 by t-test.

Due to the limited availability of reproductively old animals, we analyzed peri-gonadal adipose tissues from a cohort of reproductively young and old animals that were both part of a separate study involving superovulation. Both reproductively young and old mice received an intraperitoneal (IP) injection of 5 IU pregnant mare serum gonadotropin (PMSG, EMG Millipore, Burlington, MA) followed 44–46 h by an IP injection of 5 IU human chorionic gonadotropin (hCG, Sigma-Aldrich, St. Louis, MO), and tissue was collected 14–16 h post-hCG injection. Serum progesterone levels were not significantly different in reproductively young and old CB6F1 mice following hyperstimulation (data not shown). Mice were weighed prior to euthanasia, and adipose tissue from the peri-ovarian fat pad that surrounds the ovary was carefully dissected from both reproductively young and old mice (Fig. 1a). The isolated samples were either fixed for histochemical analysis or snap frozen for mass spectrometry (MS) and MRM-profiling as described in more detail below.

### Histochemical analysis

Tissue samples were washed in 1X phosphate buffered saline and fixed in Modified Davidson’s (Electron Microscopy Sciences, Hatfield, PA) for 16 h at 4 °C. Samples were transferred to 70% ethanol until processing, dehydrated using an automated tissue processor (Leica Microsystems), and embedded in paraffin. Sections were cut at a 5 μm thickness. Tissue sections were deparaffinized in Citrisolv (Fisher Scientific, Pittsburgh, PA) and then rehydrated in a series of graded ethanol baths (100, 70, and 30%). The deparaffinization process washes away lipids leaving behind the extracellular matrix which can be used to visualize the border of the adipocytes with a histological stain such as Picrosirus Red (PSR) which stains collagen I and III. To perform the PSR staining, slides were immersed in a staining solution prepared by dissolving Sirius Red F3B (Direct Red 80, C.I. 35,780, Sigma-Aldrich, St. Louis, MO) in a saturated aqueous solution of picric acid (Sigma-Aldrich, St. Louis, MO) at 0.1% w/v. Slides were incubated in the PSR staining solution for 40 min at room temperature followed by a wash in 0.05 M hydrochloric acid (Fisher Scientific) for 90 s. Excess acidified water was carefully wicked away from the tissue sections, and the tissue was rapidly dehydrated in 100% ethanol (3, 30 s incubations). The slides were cleared in fresh Citrisolv for five minutes and mounted using Cytoseal XYL (Fisher Scientific). All slides used for PSR staining were processed at the same time to minimize variation in staining between sequential assays.

### Adipose tissue imaging and analysis

Brightfield images were taken with an EVOS FL Auto Cell Imaging system (Thermo Fisher, Waltham, MA) using 20X or 40X objectives. To view entire adipose tissue sections, scans comprised of a series of individual images were taken across the tissue and then automatically stitched together using the EVOS software. Light settings were kept consistent between images of different tissue sections, and post-processing adjustment of brightness and contrast was done equivalently across samples to improve the clarity of the images and did not impact data analysis. Adipocytes were clearly visible due to their outline by PSR-positive staining. To quantify adipocyte number, three random areas per adipose tissue section were defined using the EVOS software, and the number of adipocytes per area was counted. We also measured the cross-sectional area of 50 adipocytes per adipose tissue section using the EVOS software, with the borders of each adipocyte defined by the PSR staining. This analysis was performed on peri-gonadal adipose tissue samples from 2 reproductively young and 3 reproductively old mice.

### Lipid extraction

For MS analysis, peri-gonadal tissue samples from 5 mice per age group were sent to the Metabolite Profiling Facility at Purdue University for MRM-profiling. The tissue from both the right and left ovaries was combined for each animal and weighed. Lipids were extracted using the Bligh & Dyer extraction method [34]. In brief, samples were transferred to a 2 mL vial with inert 1.4 mm ceramic (zirconium oxide) beads (Precellys CK 14, Bertin Corp, part # P000912-LYSK0A), and 500 μL of ultrapure water was added to homogenize the sample using the Precellys tissue homogenizer (Bertin Corp, Rockville, MD, US) at three cycles of 6200 rpm for 30 s. Next, 300 μL of homogenized tissue was transferred to a new microtube and mixed with 250 μL of chloroform and 450 μL of methanol. This solution was incubated at room temperature for 15 min. After that, 250 μL of chloroform and 250 μL of water were added and the sample was centrifuged for 10 min at 16,000 x g, forming a 2-phase solution where the bottom phase contained the lipids (organic phase). The organic phase was transferred to a new tube and dried using a speedvac centrifuge (Savant Speedvac, Thermo Scientific Inc., San Jose, CA, US), and samples were stored at -80 °C until mass spectrometry analysis.

### Targeted lipid profiling

Targeted lipid profiling was performed using discovery MRM-profiling methods and instrumentation as recently described [32]. Specifically, for sample preparation, dried lipid extracts were diluted in 50 μL of methanol/chloroform 3:1 (v/v) and 250 μL of injection solvent (acetonitrile/methanol/ammonium acetate 300 mM 3:6.65:0.35 (v/v)) to obtain a stock solution. Mass spectrometry data was acquired by flow-injection (no chromatographic separation) from 8 μL of diluted lipid extract stock solution delivered using a micro-autosampler (G1377A) to the ESI source of an Agilent 6410 triple quadrupole mass spectrometer (Agilent Technologies, Santa Clara, CA, USA). A capillary pump was connected to the autosampler and operated at a flow rate of 20 μL/min and pressure of 150 bar. Capillary voltage on the instrument was 3.5–5 kV and the gas flow 5.1 L/min at 300 °C. For the data acquisition, further dilution of the lipid extract stocks according to the sample weight was performed so that each injection corresponded to approximately 1 μg of tissue.

The comparative analysis of the amount of phosphatidylcholine (PC), sphingomyelin (SM) and triacylglycerol (TAG) lipids between old and young animals was estimated after spiking one representative sample (containing a mixture of the lipid extracts of the 5 animals) for young and one for old animals with SPLASH® LIPIDOMIX® Mass Spec Standard (Avanti Polar Lipids Inc., Alabaster, AL, US) and running the lipid profile method for these lipid classes including the MRM pair for 15:0–18:1(d7) PC, 18:1(d9) SM, and 15:0–18:1(d7)-15:0 TG for estimating the amount of PC, SM and TAG, respectively, in the two samples. Three analytical replicates were used for the data analysis.

### Statistical analysis

For the histochemical analysis, the average adipocyte area and average number of adipocytes per 10,000 μm^2^ were graphed using Graphpad Prism Software Version 8.0.1 (La Jolla, CA). Significant changes between groups were analyzed by students’ t-test and *P* values < 0.05 were considered statistically significant. For the MS analysis, relative amounts of ion abundances were used for statistics. Values of ion intensities for each of the MRMs monitored were normalized by total ion intensity of all MRMs in the method for a given sample. Further statistical analysis was then performed using Metaboanalyst 3.0 software (https://www.metaboanalyst.ca). Uploaded data was auto-scaled and volcano plots (Fold change threshold 1.5, *P* value threshold 0.05), principal component analyses (PCAs), and heatmaps (for top 25 MRMs) were plotted. Fold change was calculated by dividing values of ion intensities for each of the MRMs measured in each sample by the ion intensity of the corresponding MRM in the blank. Average fold change was graphed for significantly different lipid species using Graphpad Prism Software Version 8.0.1. Significant changes between groups were analyzed by students’ t-test and *P* values < 0.05 were considered statistically significant.

## Results

### Adipocyte size increases within peri-gonadal adipose tissue with advanced reproductive age

Advanced reproductive age was associated with increased animal weight. Reproductively old mice weighed an average of 34.8 ± 1.89 g compared to reproductively young mice which weighed 21.7 ± 0.62 g (*P* = 0.0002). This difference in total animal weight also correlated with an increase in the amount of peri-gonadal fat that surrounded the ovary that was visible by eye and also reflected in the weight of the tissue collected (Fig. 1a). We collected 195.7 ± 40.60 mg of peri-gonadal adipose tissue from reproductively old mice compared to 65.3 ± 11.79 mg in reproductively young mice (*P* = 0.015). Within the peri-gonadal adipose tissue, we noted a prominent increase in adipocyte size that occurred with advanced reproductive age (Fig. 1b-c). The average cross-sectional area of adipocytes in peri-gonadal fat from reproductively old mice was 3613.64 ± 490.51 μm^2^ compared to 1254.03 ± 368.94 μm^2^ in reproductively young mice (Fig. 1c). Consistent with this, the average number of adipocytes per area was significantly lower in peri-gonadal adipose tissue from reproductively old mice as compared to reproductively young mice (4.34 ± 1.28 adipocytes/10,000 μm^2^ vs 12.69 ± 1.64 adipocytes/10,000 μm^2^, respectively; Fig. 1d). These results suggest that advanced reproductive age is associated with adipocyte hypertrophy.

### Acyl-carnitine, phosphatidylcholine and sphingomyelin, and phosphatidylinositol lipid profiles in peri-gonadal adipose tissue cluster based on age

To determine if there were differences in lipid profiles that occur with age, we extracted lipids from peri-gonadal adipose tissue from reproductively young and old mice and performed MRM-profiling. This method enabled the interrogation of the relative amounts of numerous lipid species within ten major classes of lipids based on the LipidMaps database. These lipid classes included: triacylglycerols (TAGs), acyl-carnitines, phosphatidylcholines (PCs) and sphingomyelins (SMs), phosphatidylinositols, ceramides, cholesteryl esters, free fatty acids, phosphatidylethanolamines, phosphatidylglycerols, and phosphatidylserines. Cholesteryl ester, free fatty acid, and phosphatidylglycerol lipid profiles did not have relative ion transition measurements above background, thus preventing any further conclusions about the differences in the profiles between peri-gonadal adipose tissue from reproductively young and reproductively old mice (data not shown). No differences in ceramide, phosphatidylethanolamine, and phosphatidylserine lipid profiles were observed in peri-gonadal adipose tissue with age, and this is evident in the PCA plots for these three classes which showed minimal to no discrimination between samples from reproductively young and old mice (Additional file 2: Figure S1A-C).

**Fig. 2:**
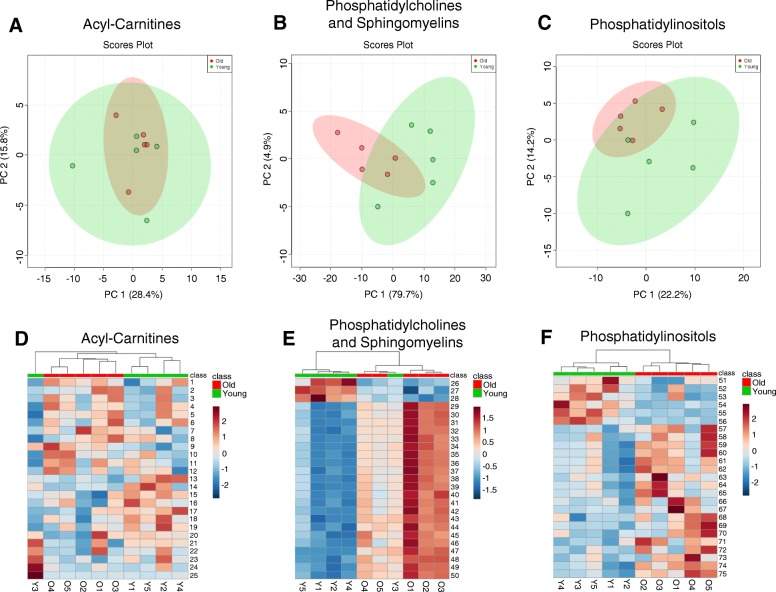
Acyl-carnitine, phosphatidylcholine (PC) and sphingomyelin (SM), and phosphatidylinositol lipid profiles in peri-gonadal adipose tissue show differences with reproductive age. 2D Principal component analysis (PCA) plots that show the separation of (**a**) Acyl-carnitines, (**b**) phosphatidylcholines (PC) and sphingomyelins (SM), and (**c**) phosphatidylinositols in peri-gonadal adipose tissue between reproductively young (green) and old (red) mice. Heatmaps representing color-coded profiling of the 25 most abundant (**d**) acyl-carnitine, (**e**) phosphatidylcholine and sphingomyelin, and (**f**) phosphatidylinositol lipid species in peri-gonadal adipose tissue from reproductively young (green) and old (red) mice. The top 25 lipid species corresponding to the numbers in the heatmaps are listed in Table 1

Although the PCA plots for acyl-carnitine, phosphatidylcholine and sphingomyelin, as well as phosphatidylinositol lipid profiles did not show significant separation between reproductively young and reproductively old mice (Fig. 2a-c), heatmaps of the 25 most abundant lipid species in each class did show clustering of samples from reproductively young and old mice (Fig. 2d-f, Table 1). This result indicated that certain lipid species in these classes are consistently present in peri-gonadal adipose tissue from reproductively young mice as compared to tissue from reproductively old mice and vice versa.

### Triacylglycerol lipid species exhibit significantly different profiles between peri-gonadal adipose tissue from reproductively young and old mice

**Fig. 3:**
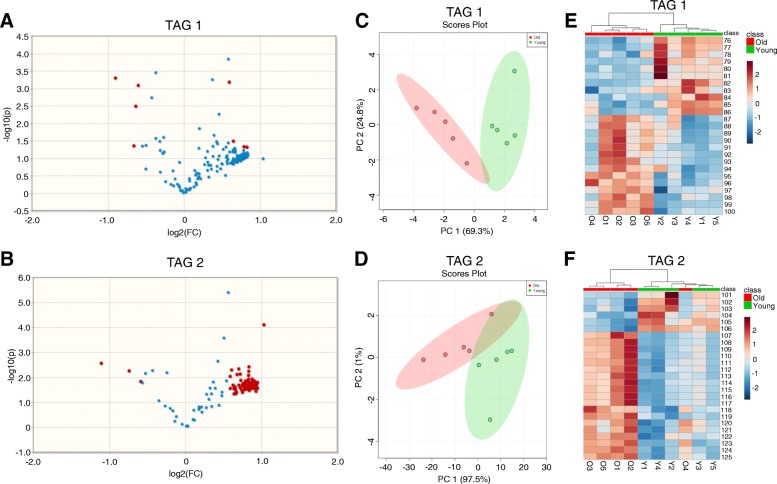
Triacylglycerol (TAG) lipid species have significantly different profiles in peri-gonadal adipose tissue from reproductively young and old mice. Volcano plots showing the log fold-change versus statistical significance (log P value) for the (**a**) Triacylglycerol (TAG) 1 and (**b**) Triacylglycerol (TAG) 2 methods. A 1.5-fold change and *p* ≤ 0.05 were used as the threshold for significance. 2D Principal component analysis (PCA) plots show the separation of triacylglycerol lipids in peri-gonadal adipose tissue samples based on age for the (**c**) TAG 1 and (**d**) TAG 2 methods. The data for reproductively young mice are shown in green and old mice are shown in red. Heatmaps representing color-coded profiling of the 25 most abundant triacylglycerol lipid species in peri-gonadal adipose tissue from reproductively young (green) and old (red) mice in the (**e**) TAG 1 method and (**f**) TAG 2 method. The top 25 lipid species corresponding to the numbers in the heatmaps are listed in Table 1

Due to the large number of triacylglycerol (TAG) lipid species interrogated in this study, TAGs were run in two separate methods (TAG 1 and TAG 2), each measuring relative ion transitions for different TAG species. The TAG species measured in each method were arbitrarily divided. This analysis revealed that 160 TAG species had significantly different profiles in peri-gonadal adipose tissue from reproductively young and reproductively old mice with a minimum fold-change of 1.5 times between the two ages, and these are highlighted in the volcano plots (Fig. 3a-b, Table 2, Additional file 1: Table S2). Seven of the 160 significantly different TAGs were more abundant in peri-gonadal adipose tissue from reproductively young mice and the remaining 153 were more abundant in tissue from reproductively old mice (Table 2, Additional file 1: Table S2). PCA plots generated based on the significantly different TAGs showed an almost completely distinct separation of the samples by age (Fig. 3c-d). Heatmaps of the 25 most abundant TAG species measured in each of the two methods showed clustering between samples from reproductively young and old mice (Fig. 3e-f, Table 1). Taken together, these results demonstrate that overall TAG lipid profiles in peri-gonadal adipose tissue differ in an age-dependent manner.

**Table 1:** The 25 most abundant lipid species in each class found in heatmaps (Figs. 2 and 3)^a^

Number on Heatmap	Lipid Name
Acyl-Carnitines	
1	3-hydroxylinoleoylcarnitine
2	O-behenoylcarnitine
3	(5Z,8Z)-3-hydroxytetradecadienoylcarnitine
4	O-adipoylcarnitine
5	(2E)-hexenedioylcarnitine, O-octanoylcarnitine
6	O-malonylcarnitine, Hydroxybutyrylcarnitine
7	Acetyl-carnitine
8	Butenylcarnitine
9	Butyrylcarnitine, Isobutyryl-L-carnitine
10	3-hydroxyarachidonoylcarnitine
11	Propenoylcarnitine
12	(9Z,12Z)-3-hydroxyhexadecadienoylcarnitine
13	Valerylcarnitine, Isovalerylcarnitine
14	Arachidyl carnitine, O-[(9Z)-17-carboxyheptadec-9-enoyl]carnitine
15	Stearidonyl carnitine
16	O-arachidonoylcarnitine
17	(9Z)-3-hydroxyoctadecenoylcarnitine
18	(9Z)-3-hydroxydodecenoylcarnitine
19	Dodecanoylcarnitine, O-dodecanoylcarnitine
20	2-Hexenoylcarnitine
21	(9Z,12Z,15Z)-3-hydroxyoctadecatrienoylcarnitine
22	Palmitoylcarnitine, (5Z)-13-carboxytridec-5-enoylcarnitine
23	(13Z,16Z)-docosadienoylcarnitine
24	Linoelaidyl carnitine, O-linoleoylcarnitine, 9,12-Hexadecadienylcarnitine
25	cis-5-Tetradecenoylcarnitine
Phosphatidylcholines and Sphingomyelins	
26	PC (32:0); PCo(33:0); PCo(32:1)OH
27	PC (36:1); PCo(37:1); PCo(36:2)OH; PC (34:3)2OH
28	SM (d18:2/24:1)
29	PC (32:3); PCo(33:3)
30	PC (42:5); PCo(42:6)OH; PC (40:7)2OH
31	PC (44:8); PC (42:10)2OH
32	SM (d18:2/18:1)
33	PC (42:0); PCo(43:0); PCo(42:1)OH; PC (40:2)2OH
34	PCo(44:4); PC (43:4)
35	PC (50:0)
36	PC (46:0)
37	PCo(40:6); PC (39:6); PC (38:7)OH; PC (36:1)2OH
38	PC (42:1); PCo(43:1); PCo(42:2)OH
39	PC (44:7); PC (42:9)2OH
40	PCo(42:1); PC (41:1); PC (40:2)OH
41	PC (44:12); PC (43:5); PC (42:6)OH; PCp(42:6)2OH
42	PCo(42:6); PC (41:6); PC (40:7)OH; PC (38:1)2OH
43	PCo(34:3)
44	PCo(32:2)
45	PCo(40:0); PC (39:0)
46	PC (52:0)
47	PCo(40:1)
48	PCo(38:5); PC (37:5); PC (34:0)2OH
49	PCo(40:5); PC (39:5); PC (38:6)OH; PC (36:0)2OH; PCp(38:6)2OH
50	PCo(38:0)
Phosphatidylinositols	
51	PI (36:2)
52	PI (12:0)
53	PI (26:0)
54	PI (20:4)
55	PI (36:7)
56	PIo (38:2), PIp (38:1); PIp (38:1); PIo (38:2)
57	PI (40:1)
58	PI (42:7)
59	PIp (42:2)
60	PI (44:1)
61	PI (42:1)
62	PI (44:10)
63	PIp (38:6)
64	PI (44:5)
65	PIo (36:1), PIp (36:0)
66	PI (36:5)
67	PI (30:1)
68	PIo (38:1), PIp (38:0)
69	PI (42:8)
70	PI (40:7)
71	PI (20:5)
72	PI (18:4)
73	PI (14:0)
74	PIp (36:5)
75	PI (42:6)
TAG 1	
76	TAG(52:3)_FA 16:0
77	TAG(52:4)_FA 16:0
78	TAG(54:4)_FA 18:0
79	TAG(52:5)_FA 16:0
80	TAG(54:5)_FA 18:1
81	TAG(54:6)_FA 18:1
82	TAG(52:2)_FA 18:0
83	TAG(54:3)_FA 16:0
84	TAG(50:0)_FA 16:0
85	TAG(50:3)_FA 16:0
86	TAG(50:2)_FA 16:0
87	TAG (49:7)
88	TAG(48:1)_FA 18:0
89	TAG(54:8)_FA 16:1
90	TAG(56:8)_FA 16:1
91	TAG(56:2)_FA 18:0
92	TAG(53:7)
93	TAG(54:8)_FA 18:1
94	TAG(54:6)_FA 16:1
95	TAG(52:2)_FA 16:1
96	TAG(52:3)_FA 16:1
97	TAG(52:2)_FA 18:1
98	TAG(54:1)_FA 18:0
99	TAG(54:3)_FA 18:1
100	TAG(54:2)_FA 18:1
TAG 2	
101	TAG(54:7)_FA 18:2
102	TAG(54:6)_FA 18:2
103	TAG(54:5)_FA 18:2
104	TAG(50:2)_FA 18:2
105	TAG(50:3)_FA 18:2
106	TAG(52:4)_FA 18:2
107	TAG(56:5)_FA 20:4
108	TAG(56:2)_FA 20:0
109	TAG(58:10)_FA 18:2
110	TAG(58:7)_FA 18:2
111	TAG(56:8)_FA 18:1
112	TAG(58:9)_FA 18:2
113	TAG(58:9)_FA 18:1
114	TAG(52:2)_FA 20:0
115	TAG(58:5)_FA 18:1
116	TAG(58:6)_FA 20:4
117	TAG(54:8)_FA 20:4
118	TAG(56:5)_FA 18:1
119	TAG(56:2)_FA 18:1
120	TAG(52:5)_FA 20:4
121	TAG(52:6)_FA 20:4
122	TAG(54:7)_FA 20:4
123	TAG(56:6)_FA 20:4
124	TAG(58:8)_FA 18:1
125	TAG(56:7)_FA 18:1

^a^*Lipid names are listed as 2 or 3 letters for the class abbreviations followed by the number of carbon, a colon, and the number unsaturation in the fatty acyl chains (*e.g. *PC(32:3). PC- phosphatidylcholine, PCo - PC lipid with an alkyl ether substituent; PI - phosphatidylinositol, PIo: PI lipid with an alkyl ether substituent, PIp: PI lipid with a 1Z-alkenyl ether (plasmalogen) substituent, SM - sphyngomyelin, TAG - triacylglycerol. The TAG lipids were profiled using a product ion related to one of the fatty acyl chains since this lipid class has no polar head as the phospholipids or another specific functional group. The targeted fatty acyl chain is indicated after the TAG abbreviation by _FA carbon number:unsaturation (*e.g. *TAG(52:3)_FA 16:0*

**Fig. 4:**
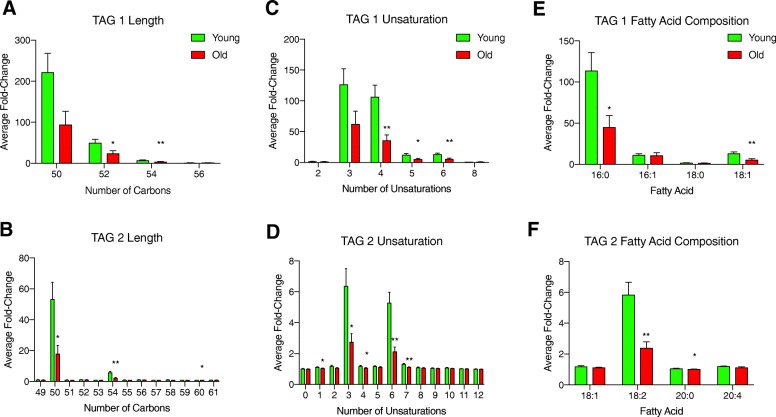
Characteristics of the TAG species that change significantly with age in peri-gonadal adipose tissue. The average fold-change with regards to blank for TAG species by length (number of carbons) in peri-gonadal adipose tissue from reproductively young (green) and old (red) mice in the (**a**) TAG 1 method and (**b**) TAG 2 method. The average fold-change over blank for TAG species by number of unsaturation sites in peri-gonadal adipose tissue from reproductively young (green) and old (red) mice in the (**c**) TAG 1 method and (**d**) TAG 2 method (**e**). The average fold-change over blank for TAG species based on fatty acid composition in peri-gonadal adipose tissue from reproductively young (green) and old (red) mice in the (**f**) TAG 1 method and (**g**) TAG 2 method. Fatty acids are labeled as the length (number of carbons): number of unsaturation sites. Error bars represent standard error between samples. *P value ≤0.05 and **P value ≤0.01 by t-test

To further characterize the 160 TAGs that were significantly different between reproductively young and reproductively old mice, we analyzed them based on length, unsaturation, and fatty acid composition. When comparing TAGs based on the number of carbons, there were several where the average fold change relative to ion transition measurements taken from a blank sample were decreased in peri-gonadal adipose tissue from reproductively old mice compared to reproductively young mice (Fig. 4a-b). In the TAG 1 method, there was a significant decrease in average fold change in TAGs with 52 and 54 carbons, and in the TAG 2 method, there was a significant decrease in TAGs with 50, 54, and 60 carbons (Fig. 4a-b). In addition to changes in the length of the fatty acid chains, there were also changes in saturation with age (Fig. 4c-d). In the TAG 1 method, the average fold change was significantly less for TAGs that had 4, 5, or 6 sites of unsaturation in peri-gonadal adipose tissue from reproductively old mice compared to young mice (Fig. 4c). The TAG 2 method showed a significant decrease in average fold-change for TAGs with 1, 3, 4, 6, and 7 sites of unsaturation with advanced reproductive age (Fig. 4e). In the TAG 1 method, there was a significant decrease in average fold-change in TAGs containing palmitic acid (16:0) and stearic acid (18:0) in peri-gonadal adipose tissue from reproductively old mice compared to young mice. (Fig. 4e). In the TAG 2 method, a similar decrease with age was observed in the average fold change for TAGs containing linoleic acid (18:2) and arachidic acid (20:0) (Fig. 4f). These results further underscore the differences in lipid profiles that occur with age in peri-gonadal adipose tissue.

### The amount of TAGs per tissue mass quantitatively decreases with age in peri-gonadal adipose tissue

**Fig. 5:**
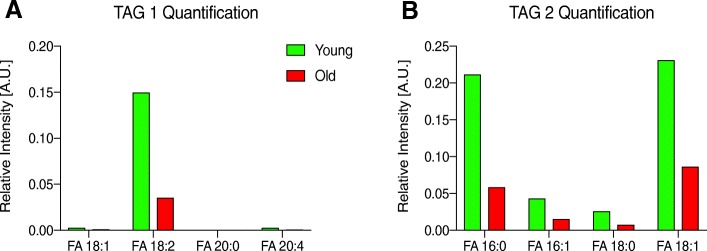
Peri-gonadal adipose tissue from reproductively young mice has quantitatively more TAGs per milligram of tissue than reproductively old mice. The average fold-change with regards to blank for TAG species by fatty acid composition in peri-gonadal adipose tissue from reproductively young (green) and old (red) mice in the (**a**) TAG 1 method and (**b**) TAG 2 method. Fatty acids are labeled as the length (number of carbons): number of unsaturation sites

To examine whether the changes in lipid profiles that occur with age are accompanied by quantitative differences in the amounts of the lipids, we compared relative ion transition measurements collected from MRM-profiling of peri-gonadal adipose tissue from reproductively young and old mice to those collected from standards of known concentrations for a given lipid class. We performed this analysis for the TAGs as well as PCs and SMs (Table 3). The PCs and SMs were present in similar quantities in peri-gonadal adipose tissue from reproductively young and old mice, and this conservation across age may be expected given the structural importance of this lipid class to cell membranes. However, the quantity of TAGs decreased with advanced reproductive age from 33.1 μg per mg of adipose tissue in reproductively young mice to 10.2 μg per mg of adipose tissue in reproductively old mice (Table 3). Consistent with this result, summed relative intensities of TAGs containing linoleic (18:2), palmitic (16:0), palmitoleic (16:1), stearic (18:0), and oleic (18:1) fatty acid residues also decreased with advanced reproductive age (Fig. 5).

**Table 2:** The number of significantly different TAGs in peri-gonadal adipose tissue based on age

Lipid Subclass	Young Peri-Gonadal Adipose Tissue	Old Peri-Gonadal Adipose Tissue
TAG 1	4	4
TAG 2	3	149

**Table 3:** The amount of lipid per milligram of peri-gonadal adipose tissue in defined classes

	Young Peri-Gonadal Adipose Tissue	Old Peri-Gonadal Adipose Tissue
μg PC per mg/sample	451.2	455.7
μg SM per mg/sample	7.5	7.5
μg TAG per mg/sample	33.1	10.2

## Discussion

In this study, we expanded our knowledge of female reproductive aging by examining how the composition of the adipose tissue surrounding the ovary changes with age. This understanding is significant because fat is one of the largest organs in the body, and this specific depot comprises the local environment of the ovary in the mouse and may, therefore, influence ovarian function on the basis of proximity. Consistent with previous studies, one of the most striking observations we made was the increase in the size of the adipocytes in peri-gonadal adipose tissue with advanced reproductive age. A similar enlargement of adipocytes was observed in epididymal white adipose tissue (WAT) in middle age Wistar rats and also in epididymal and subcutaneous WAT of aged mice [35, 36]. This adipocyte hypertrophy could be a result of the limited plasticity of adipose tissue that occurs during aging [19]. In fact, the inability to recruit pre-adipocytes into the adipogenic lineage results in a failure of the tissue to expand and results in the hypertrophy of existing adipocytes [19]. It will, therefore, be informative to determine if the function of pre-adipocytes in peri-gonadal adipose tissue is compromised in mice of advanced reproductive age.

Adipocyte size is also related to the expression of cytokines, with hypertrophic cells favoring secretion of proinflammatory adipokines [16]. Interestingly, adipose tissue is a major site of accumulation of senescent cells with age. Cellular senescence occurs when cells enter a permanent cell cycle arrest due to stress, DNA damage, and/or potent mitogenic signals [37]. Because of the cumulative exposure to such stimuli with age, senescent cells accumulate in aging tissues and can actually fuel the aging process through acquisition of the Senescence Associated Secretory Phenotype (SASP) [21, 38, 39]. The SASP consists of secretion of cytokines, chemokines, growth factors, and proteases, which can cause chronic sterile inflammation and disrupt surrounding tissue structure and function [21, 38, 39]. Interestingly, TNFα and interleukin-6 (IL-6) are pro-inflammatory cytokines with high expression in senescent cells in rodent adipose tissue and also in ovarian tissue from mice of advanced reproductive age [13, 16, 20]. Senescence can spread across cells, so it will be interesting to determine what inflammatory factors the peri-gonadal adipose tissue is producing and secreting and whether there is a connection between the peri-gonadal adipose tissue and the ovary [20].

In addition to increased adipocyte size, we also noted distinct alterations in the lipid composition of peri-gonadal adipose tissue with advanced reproductive age with respect to acyl-carnitines, phosphatidylcholines and sphingomyelins, phosphatidylinositols, and TAGs. The most robust age-associated differences were observed in the TAGs. A major role of fat is to store calorically-rich fatty acids. However, because fatty acids are reactive and cytotoxic molecules, cells store fatty acids as less reactive triglycerides. With age, however, there is a reduction in the incorporation of fatty acids as triglycerides [14]. This is consistent with our observation that there were quantitatively fewer TAGs per mg of peri-gonadal adipose tissue in reproductively old mice relative to young controls. The inability to store lipid can lead to the release of toxic free fatty acids that are then deposited into ectopic sites such as the liver, muscle, and pancreas, leading to lipotoxicity and organ damage [16, 19, 20]. Defining whether there is dysregulation of fatty acid storage and release in the peri-gonadal adipose tissue in reproductively old female mice warrants further investigation. This may in fact be the case, however, because we noted an increase in hepatic triglycerides in livers from mice of advanced reproductive age compared to controls (unpublished data). Additional quantitative MS studies will also be required to determine what lipid species may be replacing the TAGs in the peri-gonadal adipose tissue with age that may account for the observed cell hypertrophy.

Age-associated changes in adipose tissue may have important ramifications as it relates to the ovary. For example, peri-ovarian tissue can regulate ovarian function in mice. Surgical removal of peri-ovarian tissue results in several phenotypes including delayed antral follicle growth, increased atretic follicle growth, and alterations in adipokines, growth factors, lipid accumulation, and expression of steroidogenic enzymes [40]. Thus, it is highly likely that age-associated changes in the peri-gonadal tissue could impact follicle dynamics and the ovarian environment. In addition, our findings may have clinical implications. Recent ovarian transplantation studies in mouse and xenotransplantation models have demonstrated that addition of adipose tissue-derived stem cells can improve the function of ovarian grafts as measured by the increase in the total volume of the ovary, progesterone and estradiol production, and number of follicles [41–46]. This improved function is thought to be due to factors that the adipose-derived mesenchymal stromal cells produce, including pro-angiogenic cytokines and anti-apoptotic and anti-inflammatory factors. These factors protect tissues against ischemia-reperfusion damage, which is the primary limitation of ovarian grafts [41–46]. Our findings are significant within this context because if co-transplantation of adipose tissue-derived stem cells and ovarian tissue is used clinically for ovarian tissue transplantation to restore endocrine function and/or fertility in women who have undergone gonadotoxic therapies, the age of the adipose tissue donor will be a critical variable to consider due to potential variations in adipose tissue architecture and composition.

There are several caveats surrounding our data which are important to consider. First, we do not know if the age-related changes in adipocyte architecture and lipid profiles we observed are specific to the peri-gonadal fat or to the adipose tissue in general. Second, humans do not have peri-gonadal fat per se, so whether analogous changes are conserved in omental adipose tissue will be important to investigate. The visceral omental fat most proximal to the ovary may influence reproductive function, and there is a precedent that dysfunctional adipose can negatively impact extra-adipose tissues and contribute to disease and possibly aging tissues. For example, inflammatory changes in adipose tissue and metabolic dysregulation observed in animal models of alcoholic liver disease (ALD) and obesity are associated with liver steatosis and inflammation [47–49]. Finally, more than 50% of dietary fat is stored in subcutaneous and visceral fat, with visceral fat being more efficient at storage [14]. Thus, some of the lipid changes we detected may also be influenced by diet. Our reproductively young and old mouse cohorts originated from different vendors and were initially fed different chows. However, the slight variations in fat content and fatty acid composition in the chows do not appear to correlate with the age-associated changes in peri-gonadal fat lipid profiles. Therefore, it appears that the diet effects are likely minor relative to the age effects. It is important to recognize, however, that diet can affect ovarian function. For example, low protein intake reduces mouse primordial follicle activation, and omega-3 polyunsaturated fatty acids supplements alter mouse follicular cell gene expression [50, 51]. Thus, systematically determining the effect of specific diets on periovarian adipose tissue architecture and lipid profiles could be informative.

Although our primary interest is reproductive aging, we cannot overlook the interplay between aging and obesity. The reproductively old mice in our study weighed significantly more than our reproductively young mice as has been documented in other strains as well [12]. This weight increase was also accompanied by a larger mass of peri-gonadal fat with advanced reproductive age. These findings are consistent with what occurs in the human population with age where it is well-established that fat tissue mass and visceral adipose deposition increases during middle age [17, 20]. There are several parallels between obesity and aging, including enhanced chronic inflammation, circulation of proinflammatory factors, ectopic fat deposition, and lipotoxicity [16, 20, 52]. Obesity also accelerates diseases common to old age. Interestingly, there are well-characterized effects of obesity on gamete quality and fertility in both preclinical and clinical settings [53, 54]. For example, mouse models of obesity produce poor quality oocytes with aberrant maternal gene expression, dysfunctional mitochondria, and meiotic errors [55, 56]. In females undergoing Assisted Reproduction Technology cycles, oocyte and embryo counts per in vitro fertilization cycle are significantly lower in women with a body mass index above the normal range, especially in women with class-II/III obesity [57]. Given these parallels, it will be important to determine whether aging and obesity effects on reproductive function operate via independent or compounding mechanisms.

## Conclusions

These findings provide clear evidence of age-associated physiological changes in peri-gonadal adipose tissue with regards to changes in adipocyte morphology and lipid profiles. Further investigation of the effect of peri-gonadal adipose tissue on the ovarian microenvironment may identify it as a promising target to improve reproductive function in the context of age-related infertility. For example, if aging fat tissue negatively impacts reproductive function, adipose transplantation or resection may be effective therapeutic strategies.

## Additional files

Additional file 1: **Table S1.** Fat macronutrient content and fatty acid composition of mouse chows . **Table S2.** List of 160 TAGs that were significantly different with age^1^. (DOCX 30 kb)

Additional file 2: **Figure S1.** Ceramide, phosphatidylethanolamine, and phosphatidylserine lipid profiles are not different between peri-gonadal adipose tissue from reproductively young and old mice. 2D Principal component analysis (PCA) plot analysis of the lipid distribution between peri-gonadal adipose tissue from reproductively young (green) and old (red) mice for (A) ceramides, (B) phosphatidylethanolamines, and (C) phosphatidylserines. (DOCX 68 kb)
