# Supplementary material for: Histologic analysis and lipid profiling reveal reproductive age-associated changes in peri-ovarian adipose tissue
Source: Reprod Biol Endocrinol. 2019 Jun 12;17:46. doi: 10.1186/s12958-019-0487-6 (PMC6563378; doi:10.1186/s12958-019-0487-6)
Supplement: Supplementary file 1 — Table S1. Fat macronutrient content and fatty acid composition of mouse chows . Table S2. List of 160 TAGs that were significantly different with age1. (DOCX 30 kb) [file 12958_2019_487_MOESM1_ESM.docx]

| **Mouse cohort (site)** | **Young Mice**  **(Envigo)    ​** | **Old Mice**  **(NIA)** | **​Young and Old Mice**  **(Northwestern)** |
| --- | --- | --- | --- |
| **​Chow**  **(source)** | ​Teklad Global 2018  (Envigo) | ​*LabDiet* 5L79  (Charles River) | ​Teklad Global 2016  (Envigo) |
| **​Macronutrient**  **(% fat)** | ​6.2 | ​5.7 | ​4.0 |
| **​% calories**  **(from fat)** | ​18 | ​14.725 | ​12 |
| **​**  **Fatty acids** | C16:0 Palmitic % 0.7  C18:0 Stearic % 0.2  C18:1ω9 Oleic % 1.2  C18:2ω6 Linoleic % 3.1  C18:3ω3 Linolenic % 0.3  Total Saturated % 0.9  Total Monounsaturated % 1.3  Total Polyunsaturated % 3.4 | ​Fat (acid hydrolysis) % 6.8  Linoleic Acid % 1.6  Linolenic Acid % 0.1  Arachidonic Acid % <0.1  Omega-3 Fatty Acids % 1.8  Total Saturated % 1.8  Total Monounsaturated % 1.9 | ​C16:0 Palmitic % 0.5  C18:0 Stearic % 0.1  C18:1ω9 Oleic % 0.7  C18:2ω6 Linoleic % 2.0  C18:3ω3 Linolenic % 0.1  Total Saturated % 0.6  Total Monounsaturated % 0.7  Total Polyunsaturated % 2.1 |

**Supplemental Table 1. Fat macronutrient content and fatty acid composition of mouse chows**

**Supplemental Table 2. List of 160 TAGs that were significantly different with age^1^**

| TAG 1 | | |
| --- | --- | --- |
| Lipid | **Fold change** | **p-value** |
| TAG(50:3)_FA 16:0 | 0.65907 | 0.00081943 |
| TAG(52:3)_FA 16:1 | 1.5123 | 0.00066364 |
| TAG(52:4)_FA 16:0 | 0.53524 | 0.00050605 |
| TAG(52:5)_FA 16:0 | 0.64422 | 0.0032604 |
| TAG(54:6)_FA 18:1 | 0.6335 | 0.044486 |
| TAG(54:8)_FA 16:1 | 1.7299 | 0.047154 |
| TAG(56:2)_FA 18:0 | 1.5697 | 0.032589 |
| TAG(56:8)_FA 16:1 | 1.7779 | 0.048474 |
| TAG 2 | | |
| Lipid | **Fold Change** | **p-value** |
| TAG(49:7) | 1.8877 | 0.020882 |
| TAG(49:8) | 1.5469 | 0.020653 |
| TAG(50:3)_FA 18:2 | 0.59931 | 0.0055321 |
| TAG(51:8) | 1.7913 | 0.02505 |
| TAG(52:0)_FA 20:0 | 1.7808 | 0.015189 |
| TAG(52:1)_FA20:0 | 1.7204 | 0.028982 |
| TAG(52:2)_FA 20:0 | 1.7324 | 0.014359 |
| TAG(52:3)_FA 20:0 | 1.653 | 0.026279 |
| TAG(52:4)_FA 20:0 | 1.5714 | 0.024196 |
| TAG(52:5)_FA 20:0 | 1.9323 | 0.016138 |
| TAG(52:5)_FA 20:4 | 1.6301 | 0.0046027 |
| TAG(52:6)_FA 20:0 | 1.8864 | 0.018686 |
| TAG(52:6)_FA 20:4 | 1.6515 | 0.0074524 |
| TAG(53:10) | 1.7937 | 0.022263 |
| TAG(53:7) | 1.8417 | 0.019903 |
| TAG(53:7) | 1.8963 | 0.015689 |
| TAG(53:8) | 1.8515 | 0.022637 |
| TAG(53:9) | 1.8742 | 0.018867 |
| TAG(54:0)_FA 20:0 | 1.8371 | 0.024391 |
| TAG(54:3)_FA 20:0 | 1.5856 | 0.045291 |
| TAG(54:4)_FA 20:0 | 1.7746 | 0.025234 |
| TAG(54:4)_FA 20:4 | 1.5082 | 0.027977 |
| TAG(54:5)_FA 20:0 | 1.9092 | 0.018912 |
| TAG(54:6)_FA 18:2 | 0.46487 | 0.0027498 |
| TAG(54:6)_FA 20:0 | 1.8598 | 0.021443 |
| TAG(54:7)_FA 18:2 | 0.66647 | 0.014789 |
| TAG(54:7)_FA 20:0 | 1.8329 | 0.023539 |
| TAG(54:7)_FA 20:4 | 1.5067 | 0.0092002 |
| TAG(54:8)_FA 18:2 | 1.561 | 0.04633 |
| TAG(54:8)_FA 20:0 | 1.8685 | 0.022278 |
| TAG(54:8)_FA 20:4 | 1.8761 | 0.013718 |
| TAG(55:10) | 1.7697 | 0.02185 |
| TAG(55:7) | 1.8854 | 0.017992 |
| TAG(55:7) | 1.8497 | 0.024737 |
| TAG(55:8) | 1.7328 | 0.020822 |
| TAG(55:8) | 1.8772 | 0.018659 |
| TAG(55:9) | 1.8553 | 0.023456 |
| TAG(56:0)_FA 20:0 | 1.866 | 0.017223 |
| TAG(56:1)_FA 18:1 | 1.6984 | 0.017666 |
| TAG(56:1)_FA 20:0 | 1.7333 | 0.019276 |
| TAG(56:2)_FA 18:2 | 1.589 | 0.039628 |
| TAG(56:2)_FA 20:0 | 1.5263 | 0.0050674 |
| TAG(56:3)_FA 20:4 | 1.9087 | 0.023043 |
| TAG(56:4)_FA 20:4 | 1.718 | 0.023919 |
| TAG(56:5)_FA 20:0 | 1.8106 | 0.02202 |
| TAG(56:5)_FA 20:4 | 1.6036 | 0.0077215 |
| TAG(56:6)_FA 20:0 | 1.8525 | 0.022843 |
| TAG(56:7)_FA 20:0 | 1.851 | 0.027664 |
| TAG(56:8)_FA 18:1 | 1.7882 | 0.0038111 |
| TAG(56:8)_FA 20:0 | 1.8361 | 0.021093 |
| TAG(57:7) | 1.9145 | 0.018486 |
| TAG(57:7) | 1.8709 | 0.020017 |
| TAG(57:7) | 1.8683 | 0.019728 |
| TAG(57:8) | 1.8426 | 0.018509 |
| TAG(57:8) | 1.8589 | 0.022751 |
| TAG(57:9) | 1.874 | 0.021176 |
| TAG(58:0)_FA 20:0 | 1.8712 | 0.02197 |
| TAG(58:1)_FA 18:1 | 1.7634 | 0.022292 |
| TAG(58:1)_FA 20:0 | 1.8357 | 0.023429 |
| TAG(58:10)_FA 18:1 | 1.6835 | 0.019988 |
| TAG(58:10)_FA 18:2 | 1.7817 | 0.0083424 |
| TAG(58:10)_FA 20:0 | 1.8708 | 0.021966 |
| TAG(58:10)_FA 20:4 | 1.7802 | 0.025639 |
| TAG(58:2)_FA 18:1 | 1.5951 | 0.039066 |
| TAG(58:2)_FA 18:2 | 1.7951 | 0.020189 |
| TAG(58:2)_FA 20:0 | 1.8295 | 0.021107 |
| TAG(58:3)_FA 18:1 | 1.5787 | 0.030859 |
| TAG(58:3)_FA 18:2 | 1.6976 | 0.022884 |
| TAG(58:3)_FA 20:0 | 1.857 | 0.016982 |
| TAG(58:4)_FA 18:1 | 1.7094 | 0.017702 |
| TAG(58:4)_FA 18:2 | 1.5778 | 0.026109 |
| TAG(58:4)_FA 20:0 | 1.842 | 0.022002 |
| TAG(58:4)_FA 20:4 | 1.8508 | 0.023527 |
| TAG(58:5)_FA 18:1 | 1.7832 | 0.011925 |
| TAG(58:5)_FA 18:2 | 1.6737 | 0.032825 |
| TAG(58:5)_FA 20:0 | 1.8652 | 0.021959 |
| TAG(58:5)_FA 20:4 | 1.8351 | 0.017167 |
| TAG(58:6)_FA 18:1 | 1.7321 | 0.017869 |
| TAG(58:6)_FA 18:2 | 1.7902 | 0.017412 |
| TAG(58:6)_FA 20:0 | 1.8716 | 0.020603 |
| TAG(58:6)_FA 20:4 | 1.8405 | 0.011828 |
| TAG(58:7) | 1.8682 | 0.022491 |
| TAG(58:7) | 1.8889 | 0.017997 |
| TAG(58:7) | 1.886 | 0.019127 |
| TAG(58:7)_FA 18:1 | 1.5579 | 0.028296 |
| TAG(58:7)_FA 18:2 | 1.6605 | 0.013261 |
| TAG(58:7)_FA 20:0 | 1.8815 | 0.020079 |
| TAG(58:7)_FA 20:4 | 1.686 | 0.040248 |
| TAG(58:8)_FA 18:1 | 2.0488 | 7.83E-05 |
| TAG(58:8)_FA 18:2 | 1.6312 | 0.020739 |
| TAG(58:8)_FA 20:0 | 1.8487 | 0.022555 |
| TAG(58:8)_FA 20:4 | 1.8422 | 0.015152 |
| TAG(58:9)_FA 18:1 | 1.7854 | 0.0057288 |
| TAG(58:9)_FA 18:2 | 1.752 | 0.013816 |
| TAG(58:9)_FA 20:0 | 1.8594 | 0.021845 |
| TAG(58:9)_FA 20:4 | 1.8314 | 0.016542 |
| TAG(59:10) | 1.8731 | 0.020932 |
| TAG(59:8) | 1.8754 | 0.021277 |
| TAG(59:8) | 1.8655 | 0.022474 |
| TAG(59:9) | 1.8697 | 0.025301 |
| TAG(60:0)_FA 20:0 | 1.816 | 0.02571 |
| TAG(60:1)_FA 18:1 | 1.8337 | 0.022663 |
| TAG(60:1)_FA 20:0 | 1.8926 | 0.020748 |
| TAG(60:10)_FA 18:1 | 1.7612 | 0.028875 |
| TAG(60:10)_FA 18:2 | 1.8137 | 0.026145 |
| TAG(60:10)_FA 20:0 | 1.8735 | 0.0219 |
| TAG(60:10)_FA 20:4 | 1.8538 | 0.02161 |
| TAG(60:11)_FA 18:1 | 1.8847 | 0.027441 |
| TAG(60:11)_FA 18:2 | 1.7881 | 0.028304 |
| TAG(60:11)_FA 20:0 | 1.8944 | 0.017277 |
| TAG(60:11)_FA 20:4 | 1.9091 | 0.01704 |
| TAG(60:12)_FA 18:1 | 1.9005 | 0.024965 |
| TAG(60:12)_FA 18:2 | 1.8201 | 0.025568 |
| TAG(60:12)_FA 20:0 | 1.8855 | 0.019001 |
| TAG(60:12)_FA 20:4 | 1.8508 | 0.021491 |
| TAG(60:2)_FA 18:1 | 1.7476 | 0.028384 |
| TAG(60:2)_FA 18:2 | 1.8583 | 0.022053 |
| TAG(60:2)_FA 20:0 | 1.8667 | 0.021887 |
| TAG(60:3)_FA 18:1 | 1.797 | 0.016339 |
| TAG(60:3)_FA 18:2 | 1.7939 | 0.033856 |
| TAG(60:3)_FA 20:0 | 1.8551 | 0.024234 |
| TAG(60:4)_FA 18:1 | 1.7675 | 0.021285 |
| TAG(60:4)_FA 18:2 | 1.813 | 0.027065 |
| TAG(60:4)_FA 20:0 | 1.8716 | 0.020561 |
| TAG(60:4)_FA 20:4 | 1.7174 | 0.030957 |
| TAG(60:5)_FA 18:1 | 1.8755 | 0.024027 |
| TAG(60:5)_FA 18:2 | 1.7903 | 0.025466 |
| TAG(60:5)_FA 20:0 | 1.9341 | 0.027501 |
| TAG(60:5)_FA 20:4 | 1.8708 | 0.020977 |
| TAG(60:6)_FA 18:1 | 1.8639 | 0.023138 |
| TAG(60:6)_FA 18:2 | 1.8966 | 0.017966 |
| TAG(60:6)_FA 20:0 | 1.9071 | 0.036458 |
| TAG(60:6)_FA 20:4 | 1.8329 | 0.025354 |
| TAG(60:7)_FA 18:1 | 1.8372 | 0.017733 |
| TAG(60:7)_FA 18:2 | 1.8674 | 0.019736 |
| TAG(60:7)_FA 20:0 | 1.8885 | 0.019024 |
| TAG(60:7)_FA 20:4 | 1.883 | 0.019094 |
| TAG(60:8)_FA 18:1 | 1.8828 | 0.016113 |
| TAG(60:8)_FA 18:2 | 1.8483 | 0.022358 |
| TAG(60:8)_FA 20:0 | 1.8799 | 0.021797 |
| TAG(60:8)_FA 20:4 | 1.8394 | 0.022811 |
| TAG(60:9)_FA 18:1 | 1.8556 | 0.018819 |
| TAG(60:9)_FA 18:2 | 1.8112 | 0.021602 |
| TAG(60:9)_FA 20:0 | 1.9005 | 0.01955 |
| TAG(60:9)_FA 20:4 | 1.8424 | 0.022746 |
| TAG(61:10) | 1.8997 | 0.016199 |
| TAG(61:7) | 1.8687 | 0.022262 |
| TAG(61:7) | 1.8747 | 0.021135 |
| TAG(61:7) | 1.8929 | 0.025443 |
| TAG(61:8) | 1.8705 | 0.021215 |
| TAG(61:8) | 1.8729 | 0.021168 |
| TAG(61:9) | 1.8822 | 0.020531 |

^1^ *The TAG lipids were profiled using a product ion related to one of the fatty acyl chains since this lipid class has no polar head as the phospholipids or another specific functional group. The targeted fatty acyl chain is indicated after the TAG abbreviation by _FA carbon number:unsaturation (e.g. TAG(52:3)_FA 16:0.*
