# Supplementary material for: Histologic analysis and lipid profiling reveal reproductive age-associated changes in peri-ovarian adipose tissue
Source: Reprod Biol Endocrinol. 2019 Jun 12;17:46. doi: 10.1186/s12958-019-0487-6 (PMC6563378; doi:10.1186/s12958-019-0487-6)

Supplemental Figure 1

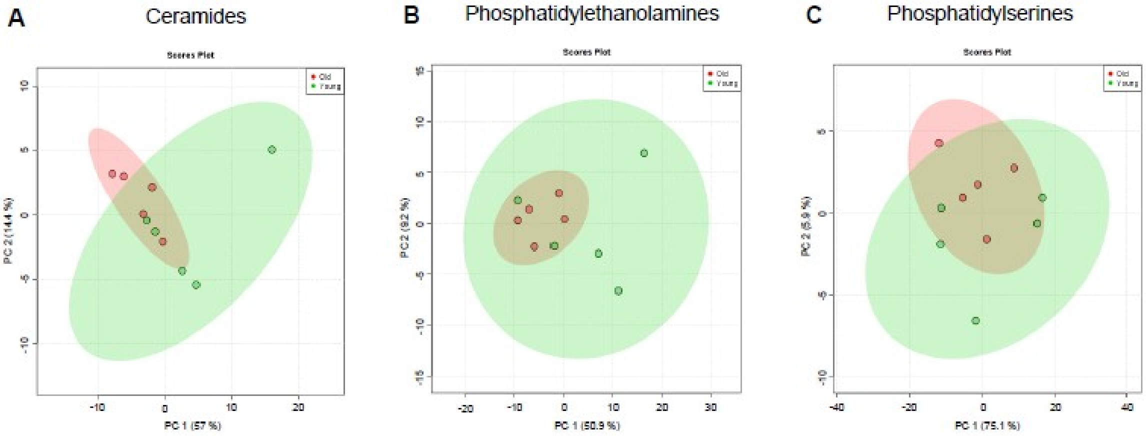

Supplement: Supplementary file 2 [file 12958_2019_487_MOESM2_ESM.docx]
